# Supplementary material for: Multiple-testing corrections in selection scans using identity-by-descent segments
Source: bioRxiv. 2025 Jan 29:2025.01.29.635528. Preprint. [Version 1] doi: 10.1101/2025.01.29.635528 (PMC11838353; doi:10.1101/2025.01.29.635528)
Supplement: 1 [file NIHPP2025.01.29.635528V1-supplement-1.pdf]

bioRxiv preprint doi: https://doi.org/10.1101/2025.01.29.635528; this version posted January 30, 2025. The copyright holder for this preprint (which was not certified by peer review) is the author/funder, who has granted bioRxiv a license to display the preprint in perpetuity. It is made available under aCC-BY 4.0 International license.

1233 # Supplementary figures

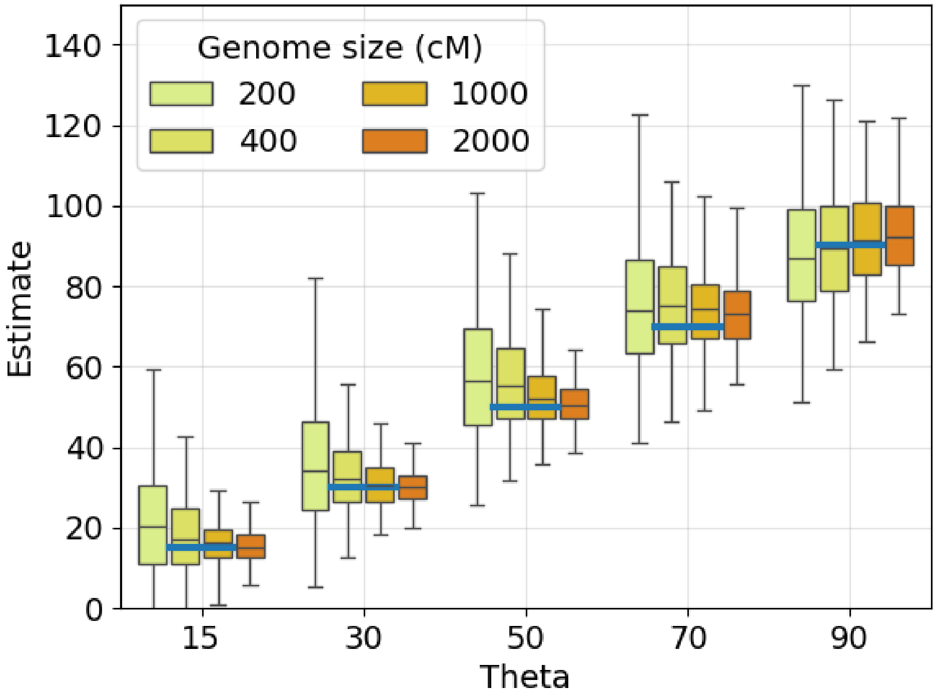

Figure S1: Estimating the exponential decay parameter θ from simulated Ornstein-Uhlenbeck processes. The 1st, 25th, 50th, 75th, and 99th percentiles of estimates $\hat{\theta}$ (y-axis) are shown for true θ (x-axis and horizontal blue lines). We estimate θ with different genome lengths (colors in legend) and step size 0.02 cM. Percentiles are taken over five hundred simulations for each θ.

bioRxiv preprint doi: https://doi.org/10.1101/2025.01.29.635528; this version posted January 30, 2025. The copyright holder for this preprint (which was not certified by peer review) is the author/funder, who has granted bioRxiv a license to display the preprint in perpetuity. It is made available under aCC-BY 4.0 International license.

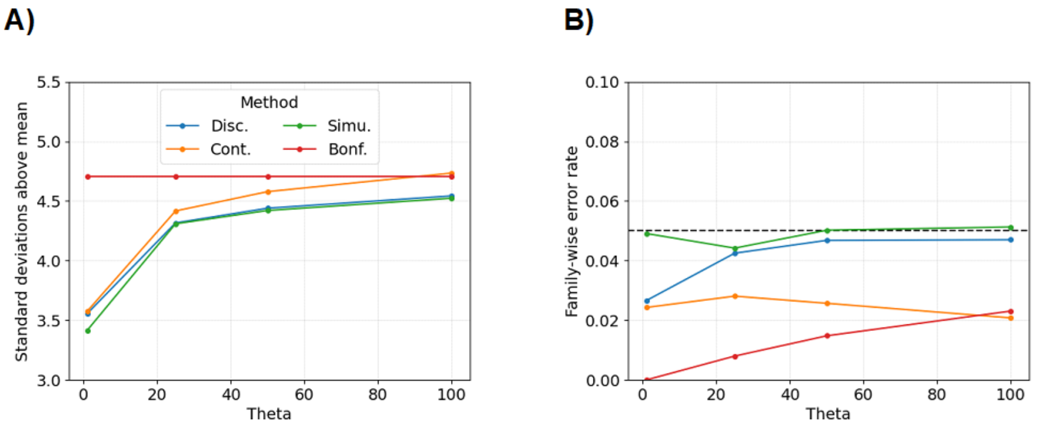

Figure S2: Multiple-testing approaches in simulations of Ornstein-Uhlenbeck processes. Line plots show A) standard deviations above the mean thresholds or B) family-wise error rates (y-axis) with different θ (x-axis). The multiple-testing approaches are (blue) the discrete-spacing analytical approximation, (orange) the continuous-spacing analytical approximation, (green) the simulation-based approach, and (red) the Bonferroni correction. The simulation-based approach is based on ten thousand simulations. The step size is hypothesis testing every 0.05 cM (50 kb). The data for each simulation is equivalent to twenty chromosomes, each of length 100 cM.

bioRxiv preprint doi: https://doi.org/10.1101/2025.01.29.635528; this version posted January 30, 2025. The copyright holder for this preprint (which was not certified by peer review) is the author/funder, who has granted bioRxiv a license to display the preprint in perpetuity. It is made available under aCC-BY 4.0 International license.

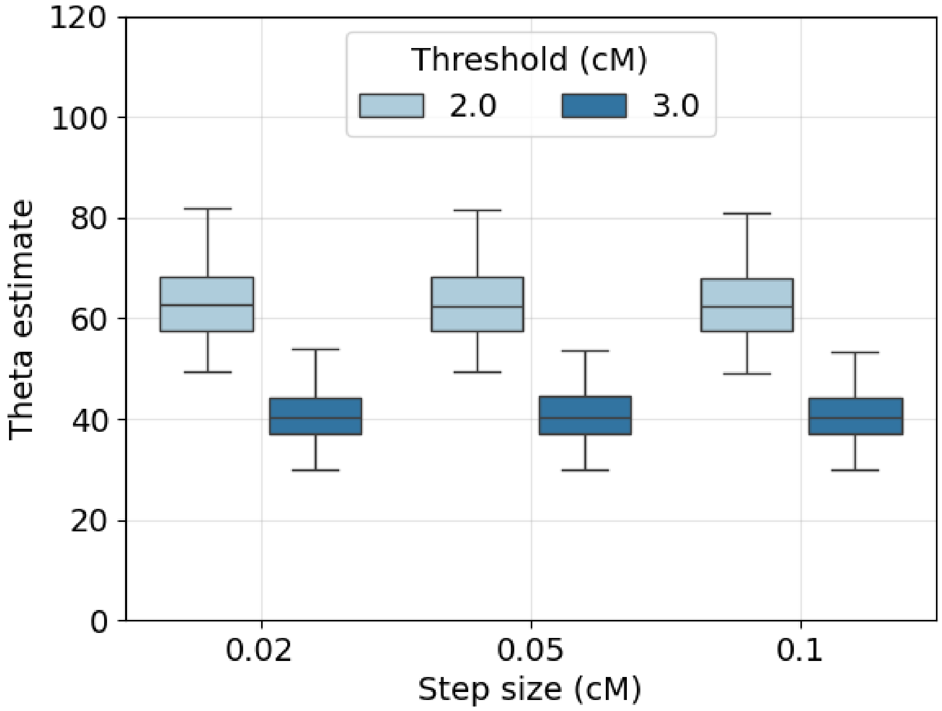

Figure S3: Estimating the exponential decay parameter θ from simulated IBD rate processes with different cM length thresholds. Box plots show the 1st, 25th, 50th, 75th, and 99th percentiles of estimates $\hat{\theta}$ using the IBD rate processes with simulated true IBD segments (dark blue) $\geq$ 2.0 cM and (light blue) $\geq$ 3.0 cM from `tskibd`. Estimates $\hat{\theta}$ are based on autocovariances calculated at different step sizes (x-axis). There are fifteen hundred simulations for each step size. The demographic model is the population bottleneck. The data for each simulation is equivalent to ten chromosomes of uniform length 100 cM.

bioRxiv preprint doi: https://doi.org/10.1101/2025.01.29.635528; this version posted January 30, 2025. The copyright holder for this preprint (which was not certified by peer review) is the author/funder, who has granted bioRxiv a license to display the preprint in perpetuity. It is made available under aCC-BY 4.0 International license.

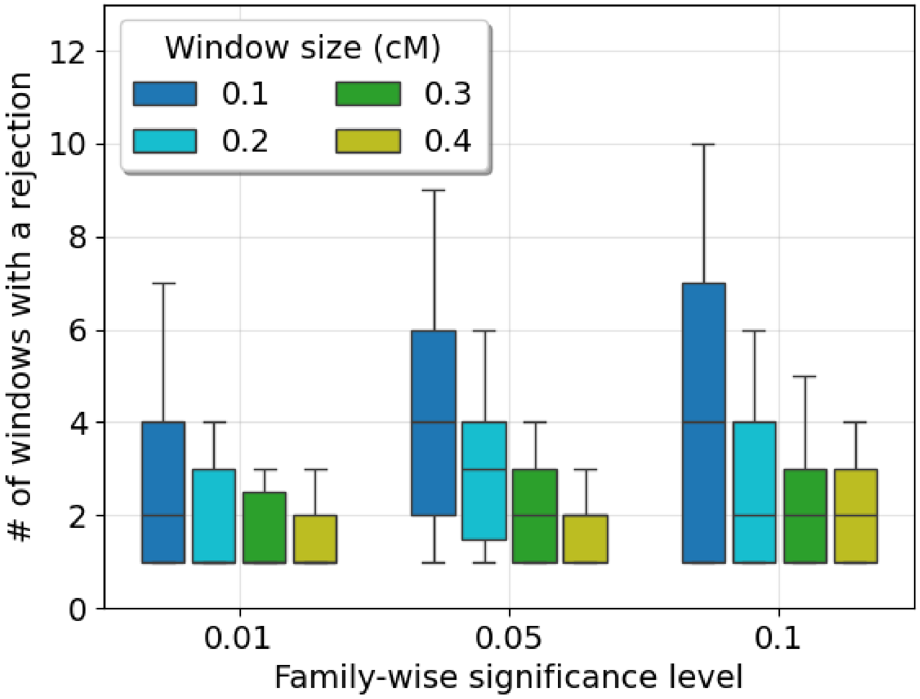

Figure S4: The number of windows with a rejected hypothesis test. Box plots show the 10th, 25th, 50th, 75th, and 90th percentiles of the number of non-overlapping windows with at least one rejection of the null hypothesis (y-axis). Windows sizes are 0.1, 0.2, 0.3, and 0.4 cM (colors in legend) with IBD rates calculated every 0.02 cM. Simulations in which there are no genome-wide significant tests are not included in the box plots. The multiple-testing method is the discrete-spacing analytical approximation using true IBD segments $\geq$ 2.0 cM. There are five hundred simulations for each family-wise significance level (x-axis). The demographic model is the population bottleneck. The data for each simulation is equivalent to ten chromosomes of uniform length 100 cM.

bioRxiv preprint doi: https://doi.org/10.1101/2025.01.29.635528; this version posted January 30, 2025. The copyright holder for this preprint (which was not certified by peer review) is the author/funder, who has granted bioRxiv a license to display the preprint in perpetuity. It is made available under aCC-BY 4.0 International license.

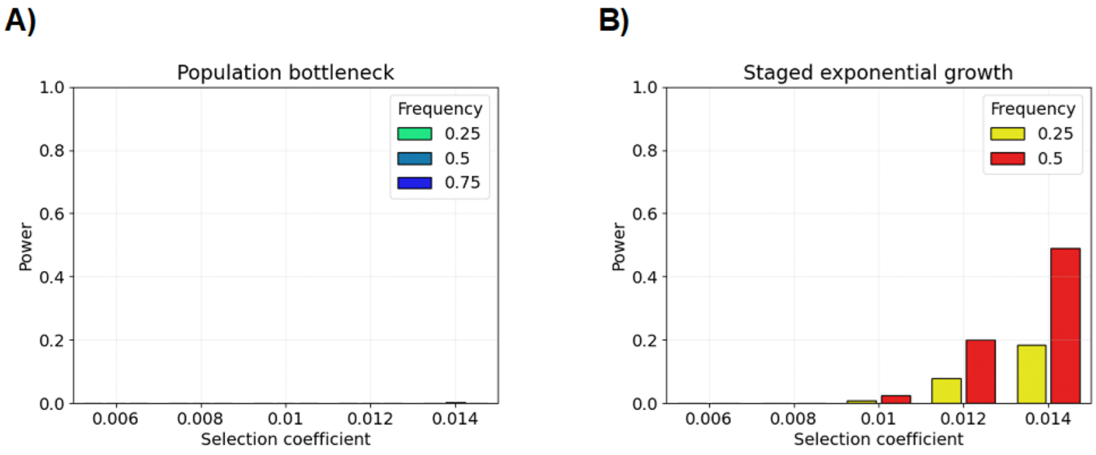

Figure S5: Power simulations for the ≥ 3.0 cM scan in different demographic models. Bar plots show the statistical power (y-axis) in the A) population bottleneck and B) staged exponential growth models using true IBD segments ≥ 2.0 cM overlapping the selected allele. Power is the proportion of tests where the null model is rejected at the p value threshold corresponding to the 0.05 family-wise significance level. Hypothesis testing is based on the discrete-spacing analytical threshold using the step size 0.02 cM. There are two hundred simulations for each pair of selection coefficient (x-axis) and current-day allele frequency (colors in legend).

bioRxiv preprint doi: https://doi.org/10.1101/2025.01.29.635528; this version posted January 30, 2025. The copyright holder for this preprint (which was not certified by peer review) is the author/funder, who has granted bioRxiv a license to display the preprint in perpetuity. It is made available under aCC-BY 4.0 International license.

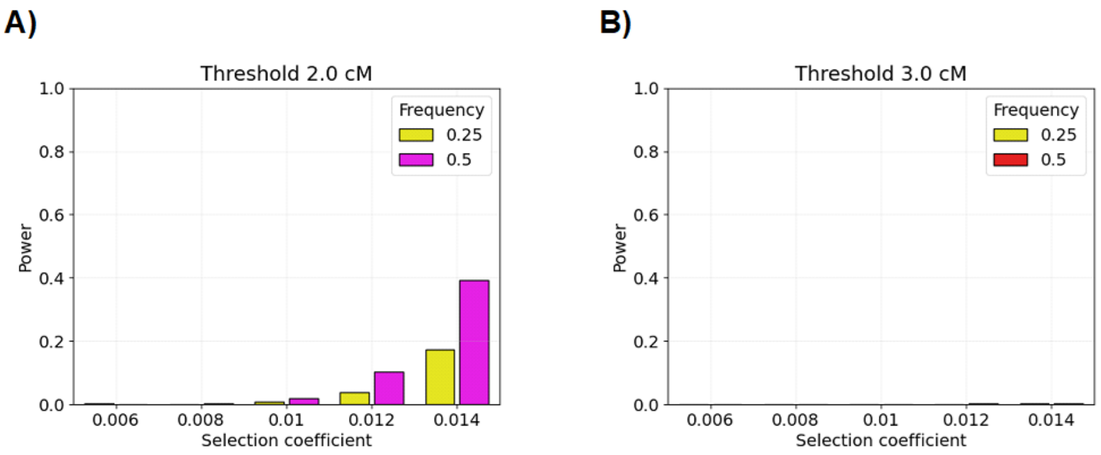

Figure S6: Power simulations in a constant size population. Bar plots show the statistical power (y-axis) using true IBD segments A) ≥ 2.0 cM or B) ≥ 3.0 cM overlapping the selected allele. Power is the proportion of tests where the null model is rejected at the p value threshold corresponding to the 0.05 family-wise significance level. Hypothesis testing is based on the discrete-spacing analytical threshold using the step size 0.02 cM. There are two hundred simulations for each pair of selection coefficient (x-axis) and current-day allele frequency (colors in legend).

bioRxiv preprint doi: https://doi.org/10.1101/2025.01.29.635528; this version posted January 30, 2025. The copyright holder for this preprint (which was not certified by peer review) is the author/funder, who has granted bioRxiv a license to display the preprint in perpetuity. It is made available under aCC-BY 4.0 International license.

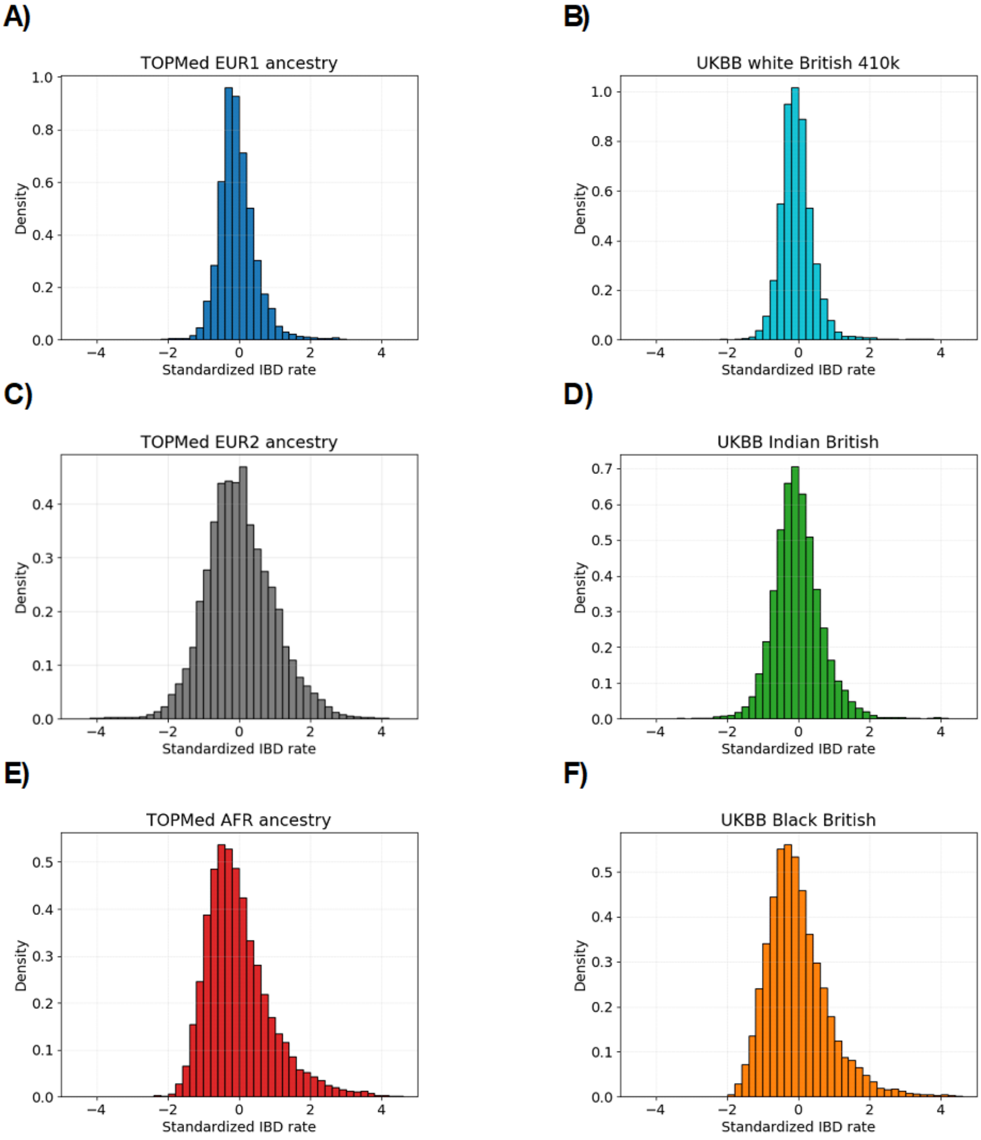

Figure S7: Histograms of IBD rates in human populations. The standardized IBD rates ≥ 2.0 cM (x-axis) are shown for A) TOPMed EUR1, B) UKBB white British, C) TOPMed EUR2, D) UKBB Indian British, E) TOPMed AFR ancestry, and F) UKBB Black British sample sets. Each histogram has fifty bins, and the x-axes range from -5 to 5.

bioRxiv preprint doi: https://doi.org/10.1101/2025.01.29.635528; this version posted January 30, 2025. The copyright holder for this preprint (which was not certified by peer review) is the author/funder, who has granted bioRxiv a license to display the preprint in perpetuity. It is made available under aCC-BY 4.0 International license.

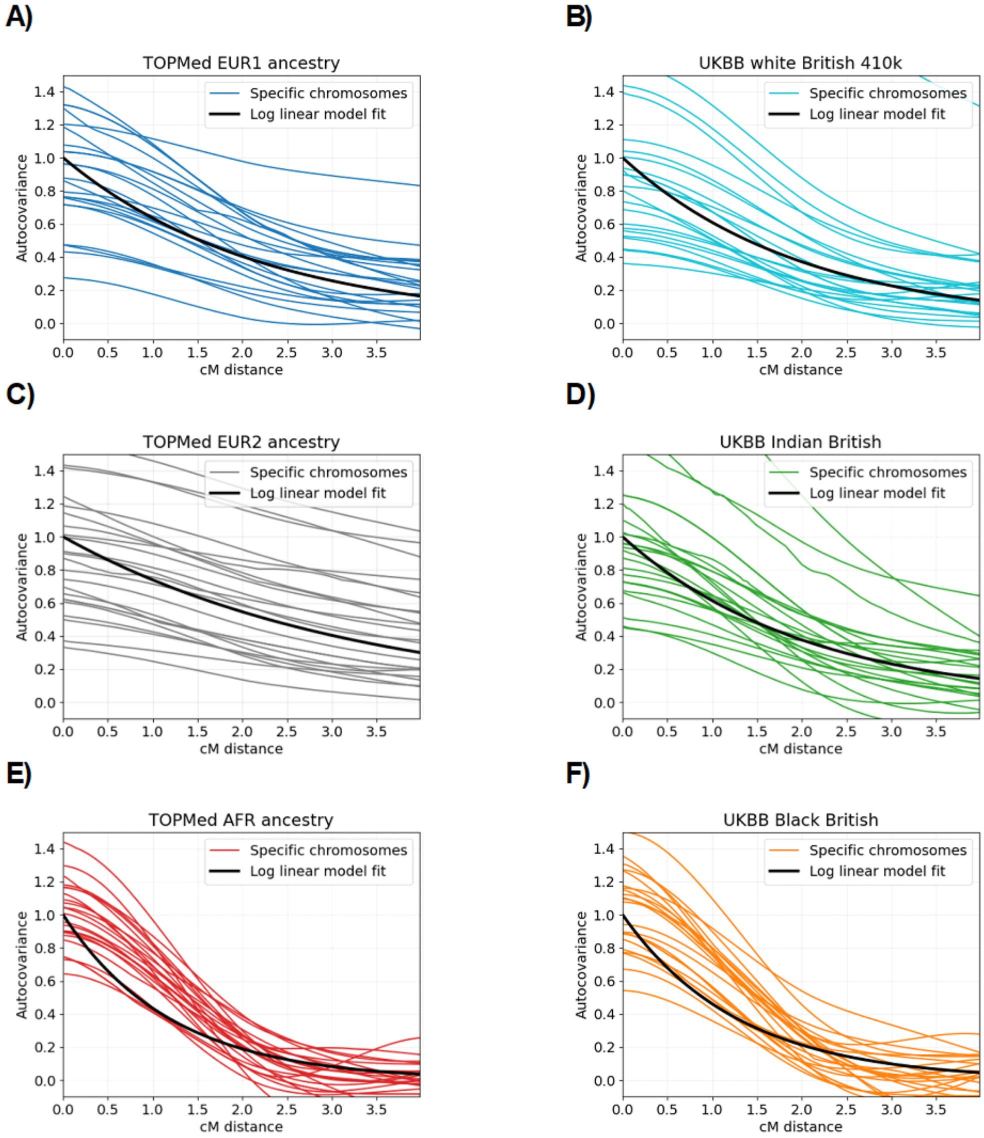

Figure S8: Estimating exponential decay parameter θ in real data. Each faint colored line shows estimated autocovariances (y-axis) for different cM distances (x-axis) and a specific chromosome. The black lines show the predicted autocovariances from the fitted Ornstein-Uhlenbeck processes using estimates $\hat{\theta}$. The data for each subplot is based on A) TOPMed EUR1 ancestry, B) UKBB white British, C) TOPMed EUR2 ancestry, D) UKBB Indian British, E) TOPMed AFR ancestry, and F) UKBB Black British sample sets.

bioRxiv preprint doi: https://doi.org/10.1101/2025.01.29.635528; this version posted January 30, 2025. The copyright holder for this preprint (which was not certified by peer review) is the author/funder, who has granted bioRxiv a license to display the preprint in perpetuity. It is made available under aCC-BY 4.0 International license.

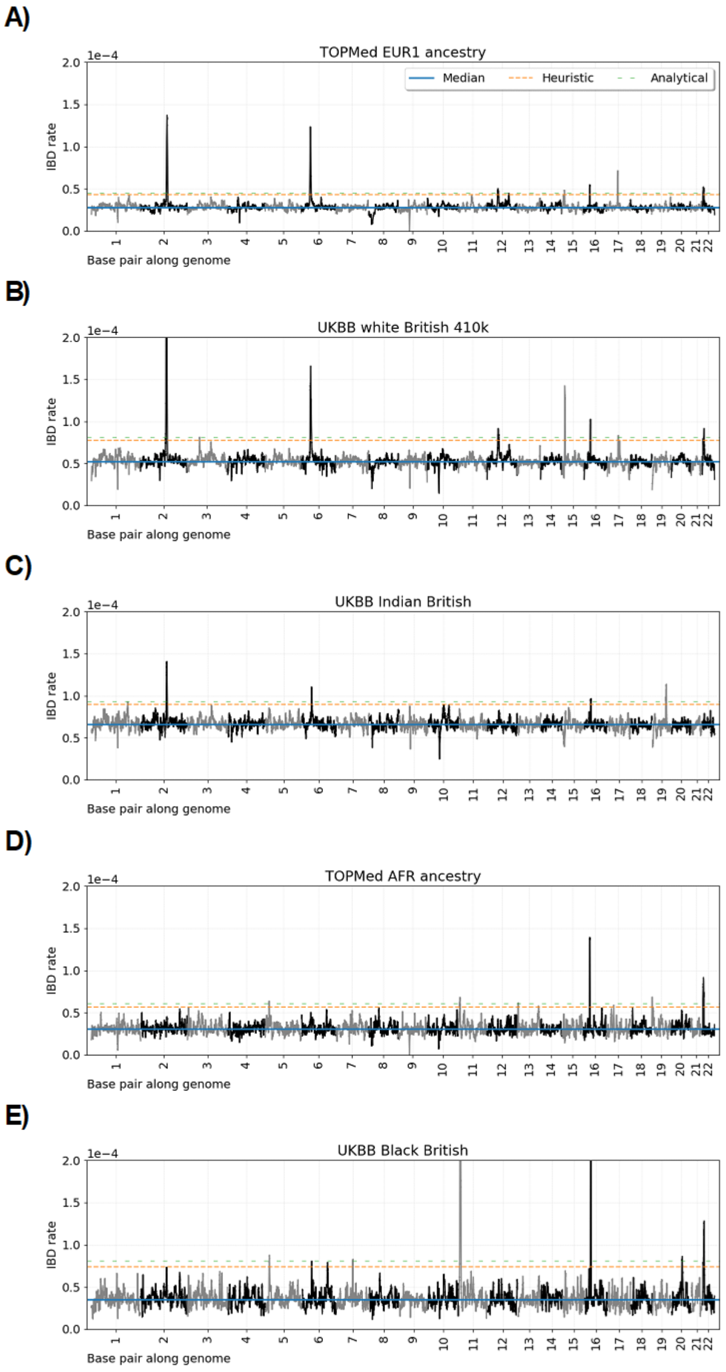

Figure S9: Genome-wide IBD rate scans using the ≥ 3.0 cM threshold. Line plots show IBD rates every 0.02 cM (y-axis) for base pair positions along twenty-two human autosomes. The data for each subplot is based on A) TOPMed EUR1 ancestry, B) UKBB white British, C) UKBB Indian British, D) TOPMed AFR ancestry, and E) UKBB Black British sample sets. Horizontal dashed lines show (blue) the genome-wide median IBD rate, (orange) the heuristic threshold of four standard deviations above the median, and (green) the analytical multiple-testing threshold.

bioRxiv preprint doi: https://doi.org/10.1101/2025.01.29.635528; this version posted January 30, 2025. The copyright holder for this preprint (which was not certified by peer review) is the author/funder, who has granted bioRxiv a license to display the preprint in perpetuity. It is made available under aCC-BY 4.0 International license.

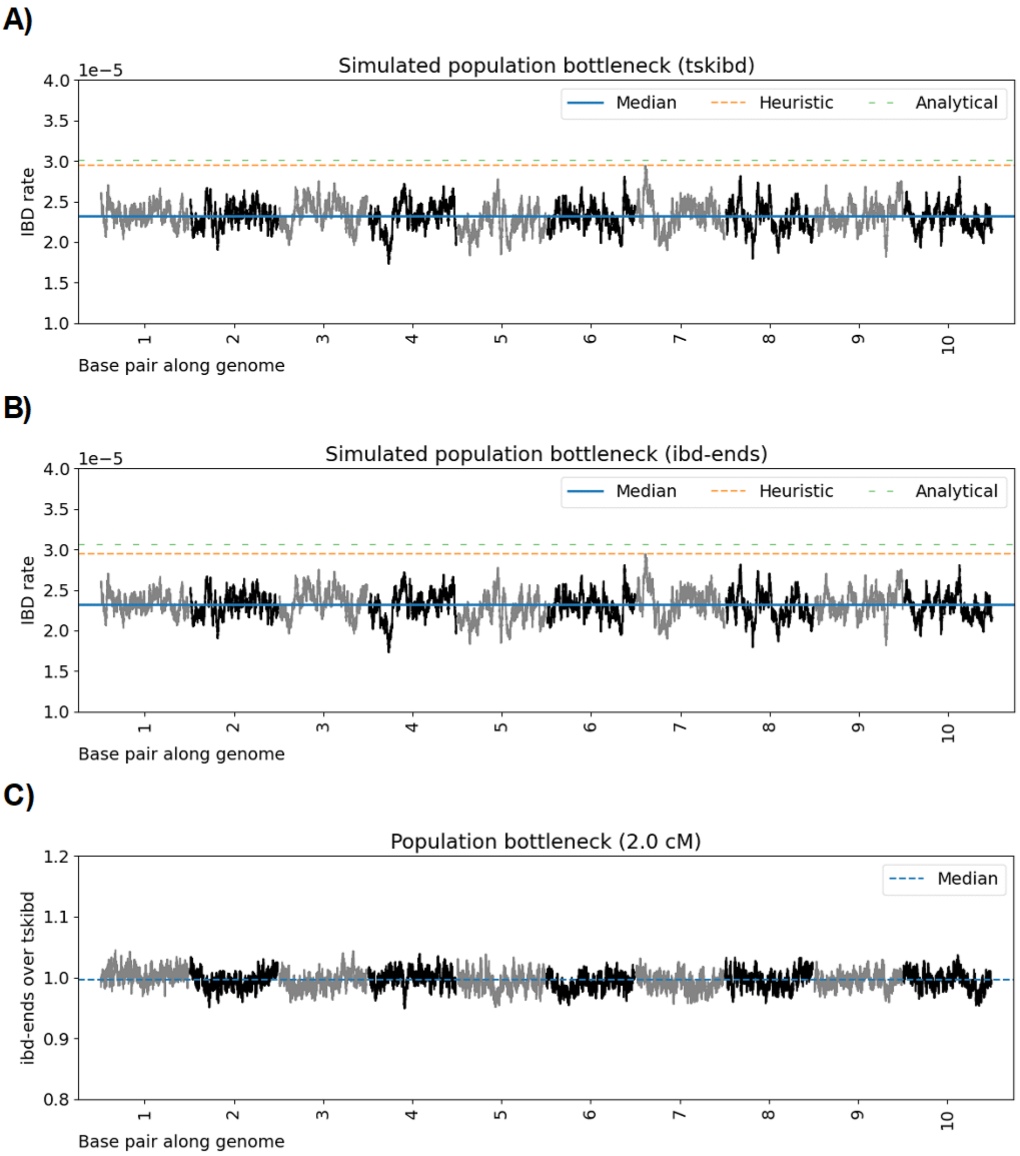

Figure S10: Genome-wide IBD rate scan in a simulated population bottleneck scenario. Line plots show $\geq$ 2.0 cM IBD rates (y-axis) for cM positions along ten simulated chromosomes. Scans are based on A) `tskibd` true IBD segments [6] or B) `ibd-ends` inferred IBD segments [24]. In C), we divide the IBD rates in B) from those in A). Each chromosome is 100 cM. The IBD rate is calculated every 0.02 cM. Data is based on twenty-five hundred diploid samples from the simulated population bottleneck demographic scenario. Horizontal dashed lines show (blue) the genome-wide median IBD rate, (orange) the heuristic threshold of four standard deviations above the median, and (green) the discrete-spacing analytical threshold). The family-wise significance level is 0.05.

bioRxiv preprint doi: https://doi.org/10.1101/2025.01.29.635528; this version posted January 30, 2025. The copyright holder for this preprint (which was not certified by peer review) is the author/funder, who has granted bioRxiv a license to display the preprint in perpetuity. It is made available under aCC-BY 4.0 International license.

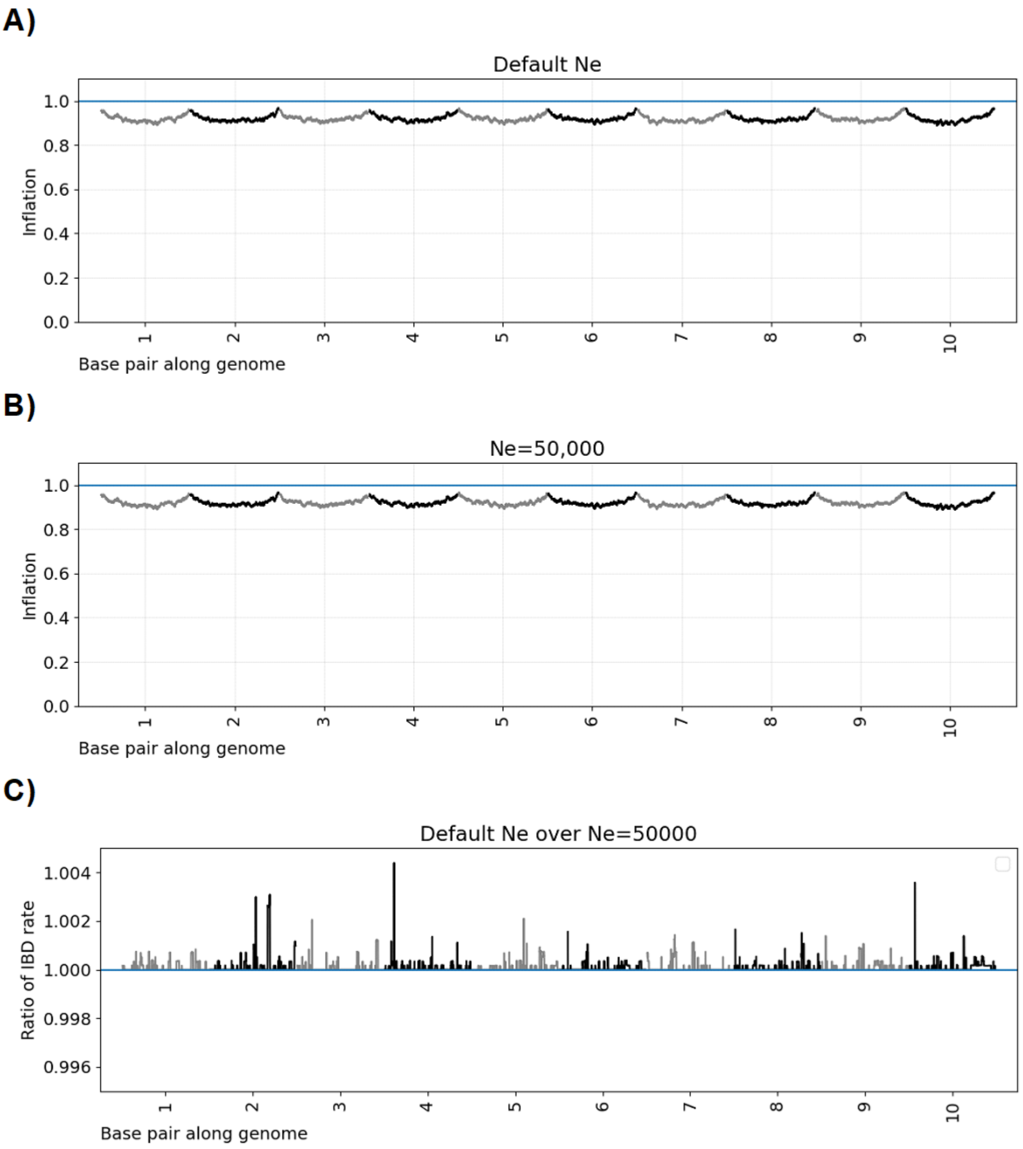

Figure S11: Genome-wide IBD rate scan in a simulated constant population size scenario. Line plots show inferred IBD rates over true IBD rates (y-axis) for cM positions along ten simulated chromosomes. Scans are based on using `ibd-ends`'s A) default `ne` setting versus B) `ne=50000`. In C), we divide the inferred IBD rates in A) and B). Each chromosome is 100 cM. The IBD rate is calculated every 0.02 cM. Data is based on twenty-five hundred diploid samples from the simulated scenario of a constant population of fifty thousand individuals. The segment detection threshold is ≥ 2.0 cM.

bioRxiv preprint doi: https://doi.org/10.1101/2025.01.29.635528; this version posted January 30, 2025. The copyright holder for this preprint (which was not certified by peer review) is the author/funder, who has granted bioRxiv a license to display the preprint in perpetuity. It is made available under aCC-BY 4.0 International license.

1234 # Supplementary tables

**Family-wise level**	**Adjusted** Analytical	Simulation	Bonferroni	**FWER** Analytical	Simulation
0.01	1.58e-6	2.01e-6	2.08e-7	0.008	0.012
0.05	9.23e-6	1.06e-5	1.04e-6	0.030	0.034
0.10	2.03e-5	2.29e-5	2.08e-6	0.066	0.078

Table S1: Significance levels and family-wise error rates after multiple-testing corrections with IBD segments $\geq$ 3.0 cM. Significance levels are adjusted for multiple testing based on scans over 10 chromosomes of size 100 cM and tests every 0.02 cM (50,000 total tests). The multiple-testing analytical and simulation-based thresholds are based on a fitted Ornstein-Uhlenbeck process. Each simulation has a different threshold as a result of estimating θ. Family-wise error rate (FWER) is the percentage of five hundred genome-wide scans with at least one statistically significant result. The demographic scenario is the population bottleneck.

bioRxiv preprint doi: https://doi.org/10.1101/2025.01.29.635528; this version posted January 30, 2025. The copyright holder for this preprint (which was not certified by peer review) is the author/funder, who has granted bioRxiv a license to display the preprint in perpetuity. It is made available under aCC-BY 4.0 International license.

Dataset	Chr	Rate (1e-4)	Region size (cM)	Position (Mb)	Genes	p value
TOPMed EUR1 (GRCh38)	2	1.37	7.94	134.84 (132.29-140.09)	*LCT*	2.59e-187
	6	1.23	8.04	31.03 (23.91-36.38)	*MHC*	2.14e-143
	17	0.71	3.44	37.68 (36.33-38.44)	*HNF1B*	2.59e-31
	16	0.54	3.00	17.74 (16.73-18.72)	*XYLT1*	8.50e-13
	22	0.52	5.12	20.23 (19.15-21.10)	.	6.37e-11
	12	0.50	5.10	51.38 (48.80-53.20)	*KRT*	1.74e-9
	15	0.48	2.28	31.30 (30.46-32.15)	*TRPM1*	4.19e-8
UKBB white British 410k (GRCh37)	2	3.98	8.72	135.91 (132.86-141.34)	*LCT*	underflow
	6	1.65	7.92	30.80 (23.91-36.34)	*MHC*	2.63e-74
	15	1.42	3.50	30.94 (30.16-32.92)	*TRPM1*	1.26e-47
	16	1.02	3.28	18.25 (16.37-18.95)	*XYLT1*	6.40e-16
	22	0.91	3.46	21.53 (20.98-21.61)	.	1.91e-10
	12	0.91	5.12	51.78 (49.38-53.76)	*KRT*	2.04e-10
	17	0.83	1.26	36.18 (35.44-36.49)	*HNF1B*	4.16e-7
UKBB Indian British (GRCh37)	2	1.41	5.12	136.97 (134.36-139.51)	*LCT*	1.69e-36
	19	1.12	4.92	50.23 (48.47-50.74)	.	5.25e-16
	6	1.10	3.12	33.96 (33.02-36.34)	*MHC*	4.70e-14
	16	0.96	2.80	18.06 (16.83-18.28)	*XYLT1*	1.65e-7
TOPMed AFR (GRCh38)	16	1.39	3.44	17.73 (16.45-19.09)	*XYLT1*	1.31e-63
	22	0.92	5.56	20.26 (18.95-21.10)	.	3.30e-21
	19	0.69	1.98	1.78 (1.72-2.10)	.	3.56e-9
	11	0.68	2.74	5.23 (3.83-5.75)	*HBB*	4.83e-9
	5	0.37	1.56	9.44 (9.20-9.72)	*SEMA5A*	2.34e-7
UKBB Black British (GRCh37)	11	3.79	7.78	4.72 (2.76-6.92)	*HBB*	2.78e-271
	16	2.52	4.14	17.40 (16.06-19.14)	*XYLT1*	4.12e-109
	22	1.28	4.74	21.54 (19.64-22.33)	.	1.68e-21
	5	0.88	1.64	9.62 (9.34-9.90)	*SEMA5A*	5.77e-8
	20	0.86	1.54	40.93 (39.47-40.99)	.	1.30e-7
	7	0.83	0.50	80.35 (80.08-80.40)	*SEMA3C*	7.61e-7

Table S2: Loci detected in the $\geq$ 3.0 cM selection scans. We report loci where identity-by-descent (IBD) rates exceed the discrete-spacing analytical thresholds of 0.45e-4, 0.81e-4, 0.93e-4, 0.61e-4, and 0.81e-4 for the TOPMed EUR1 ancestry, UKBB white British, UKBB Indian British, TOPMed AFR ancestry, and UKBB Black British sample sets, respectively. The maximum IBD rate is given for each locus. Physical positions for the location of the maximum IBD rate and the span of excess IBD rates are shown in megabases (Mb). We report the size in centiMorgan (cM) of each region, which is defined to be a contiguous stretch of IBD rates exceeding the genome-wide significance threshold. Pedigree-based recombination maps from Halldorsson et al. [80] and Bhérer et al. [82] aligned to the GRCh38 and GRCh37 reference genomes are used for inferring IBD segments in the TOPMed and UKBB sample sets, respectively. p values are calculated assuming the null model that IBD rates are normally distributed. Annotated genes or gene complexes are discussed in the main text.

bioRxiv preprint doi: https://doi.org/10.1101/2025.01.29.635528; this version posted January 30, 2025. The copyright holder for this preprint (which was not certified by peer review) is the author/funder, who has granted bioRxiv a license to display the preprint in perpetuity. It is made available under aCC-BY 4.0 International license.

Supplementary acknowledgements

We gratefully acknowledge the individual studies and participants who provided biological samples and data for the TOPMed project. Funding for the Barbados Asthma Genetics Study (BAGS) was provided by the National Institutes of Health (NIH) R01HL104608, R01HL087699, and HL104608 S1. The Mount Sinai BioMe Biobank (BioMe) has been supported by The Andrea and Charles Bronfman Philanthropies and in part by funds from the NHLBI and the National Human Genome Research Institute (NHGRI) (U01HG00638001; U01HG007417; X01HL134588); genome sequencing was funded by contract HHSN268201600037I. The Cleveland Clinic Atrial Fibrillation study (CCAF) was supported by NIH grants R01 HL 090620 and R01 HL 111314, the NIH National Center for Research Resources for Case Western Reserve University and Cleveland Clinic Clinical and Translational Science Award UL1-RR024989, the Cleveland Clinic Department of Cardiovascular Medicine philanthropy research funds, and the Tomsich Atrial Fibrillation Research Fund; genome sequencing was supported by R01HL092577. The Framingham Heart Study (FHS) was supported by contracts NO1-HC-25195, HHSN268201500001I, and 75N92019D00031 from the NHLBI and grant supplement R01 HL092577-06S1; genome sequencing was funded by HHSN268201600034I and U54HG003067. The Hypertension Genetic Epidemiology Network Study (HyperGen) is part of the NHLBI Family Blood Pressure Program; collection of the data represented here was supported by grants U01 HL054472, U01 HL054473, U01 HL054495, and U01 HL054509; genome sequencing was funded by R01HL055673. The Jackson Heart Study is supported and conducted in collaboration with Jackson State University (HHSN268201300049C and HHSN268201300050C), Touga-

bioRxiv preprint doi: https://doi.org/10.1101/2025.01.29.635528; this version posted January 30, 2025. The copyright holder for this preprint (which was not certified by peer review) is the author/funder, who has granted bioRxiv a license to display the preprint in perpetuity. It is made available under aCC-BY 4.0 International license.

loo College (HHSN268201300048C), and the University of Mississippi Medical Center (HHSN268201300046C and HHSN268201300047C) contracts from NHLBI and the National Institute for Minority Health and Health Disparities (NIMHD); genome sequencing was funded by HHSN268201100037C. The My Life, Our Future samples (MLOF) and data are made possible through the partnership of Bloodworks Northwest, the American Thrombosis and Hemostasis Network, the National Hemophilia Foundation, and Bioverativ; genome sequencing was funded by HHSN268201600033I and HHSN268201500016C. The Venous Thromboembolism project (VTE) was funded in part by grants from the NIH, NHLBI (HL66216 and HL83141), and the NHGRI (HG04735). The Vanderbilt Genetic Basis of Atrial Fibrillation study (VUAF) was supported by grants from the American Heart Association (EIA 0940116N) and grants from the National Institutes of Health (HL092217, U19 HL65962, and UL1 RR024975), and by CTSA award (UL1TR000445) from the National Center for Advancing Translational Sciences; genome sequencing was funded by R01HL092577. The Women's Health Initiative program (WHI) is funded by NHLBI through contracts 75N92021D00001, 75N92021D00002, 75N92021D00003, 75N92021D00004, 75N92021D00005; genome sequencing was funded by HHSN268201500014C.
